# Phenazine from *Pseudomonas aeruginosa* UPMP3 induced the host resistance in oil palm (*Elaeis guineensis* Jacq.)-*Ganoderma boninense* pathosystem

**DOI:** 10.1038/s41598-020-72156-7

**Published:** 2020-09-24

**Authors:** Waheeda Parvin, Nisha Govender, Radziah Othman, Hawa Jaafar, Mahbubur Rahman, Mui-Yun Wong

**Affiliations:** 1 Department of Plant Protection, Faculty of Agriculture, Universiti Putra Malaysia, Serdang, Malaysia; 2 Bangladesh Forest Research Institute, Chittagong, Bangladesh; 3 Institute of Systems Biology (INBIOSIS), Universiti Kebangsaan Malaysia, Bangi, Malaysia; 4 Department of Land Management, Faculty of Agriculture, Universiti Putra Malaysia, Serdang, Malaysia; 5 Department of Crop Science, Faculty of Agriculture, Universiti Putra Malaysia, Serdang, Malaysia; 6 Department of Biochemistry, Faculty of Biotechnology and Biomolecular Sciences, Universiti Putra Malaysia, Serdang, Malaysia; 7 Institute of Plantation Studies, Universiti Putra Malaysia, Serdang, Malaysia

**Keywords:** Plant sciences, Plant immunity, Plant molecular biology

## Abstract

*Pseudomonas aeruginosa* developed its biocontrol agent property through the production of antifungal derivatives, with the phenazine among them. In this study, the applications of crude phenazine synthesized by *Pseudomonas aeruginosa* UPMP3 and hexaconazole were comparatively evaluated for their effectiveness to suppress basal stem rot infection in artificially *G. boninense*-challenged oil palm seedlings. A glasshouse experiment under the randomized completely block design was set with the following treatments: non-inoculated seedlings, *G. boninense* inoculated seedlings, *G. boninense* inoculated seedlings with 1 mg/ml phenazine application, *G. boninense* inoculated seedlings with 2 mg/ml phenazine application and *G. boninense* inoculated seedlings with 0.048 mg/ml hexaconazole application. Seedlings were screened for disease parameters and plant vigour traits (plant height, plant fresh weight, root fresh, and dry weight, stem diameter, and total chlorophyll) at 1-to-4 month post-inoculation (mpi). The application of 2 mg/ml phenazine significantly reduced disease severity (DS) at 44% in comparison to fungicide application (DS = 67%). Plant vigour improved from 1 to 4 mpi and the rate of disease reduction in seedlings with phenazine application (2 mg/ml) was twofold greater than hexaconazole. At 4, 6 and 8 wpi, an up-regulation of *chitinase* and *β-1,3 glucanase* genes in seedlings treated with phenazine suggests the involvement of induced resistance in *G. boninense*-oil palm pathosystem.

## Introduction

Bacteria are primary producers of natural phenazines. The nitrogen containing heterocyclic phenazines are vital for the development of biofilm, also a key structure that supports the survival of bacteria under adverse environmental conditions. Phenazines function as cell signals for regulation of gene expression patterns and as electron shuttles for the modification of cellular redox states. The phenazine-producing Eubacteria includes *Burkholderia, Ervinia, Sorangium, Nocardia* and a wide variety of both gram-positive and gram-negative species^1^. A considerable number of *Pseudomonas* biocontrol strains with antifungal activity produce phenazines^2,3^. Phenazines are toxic to a wide range of microorganisms and its physical properties have been reported to influence in vivo activities at the cellular level. The antimicrobial activity of phenazine is affected by the rate of oxidative-reductive process, transformation rate of the compound and the degree at which toxic superoxide radicals are accumulated in the target cells^4^. The action of phenazine has been studied in barley, oat, rice, wheat and bean for the development of biocontrol agents targeting a wide array of phytopathogens^5,6^.

The soil-inhabitant *Pseudomonas aeruginosa* produces a number of antifungal factors such as the pyocyanin (5-n-methyl-1-hydroxy-phenazine), phenazine-1-carboxylic acid and phenazine-1-carboxamide^7–10^. These bright coloured pigments have a broad-spectrum antibiotic activity and therefore their efficiency for biocontrol applications such as biological pesticides have been extensively studied^4,11^. The axenic culture of *P. aeruginosa* UPMP3 has shown to produce core phenazine (PHZ), phenazine-l-carboxylic acid (PCA) and pyocyanin (PYO) antibiotics at variable concentrations. On an individual evaluation, each antibiotic showed in vitro antagonistic activity against *Ganoderma boninense* at a minimal inhibition ratio of 1: 10: 15/PHZ: PCA: PYO. The PHZ extracted from *P. aeruginosa* UPMP3 crude extract demonstrated the best antifungal efficiency against *Ganoderma boninense* amongst the other phenazine derivatives evaluated at a laboratory scale experiment^12^.

The basidiomycetous *G. boninense* causes basal stem rot (BSR) of oil palms (OPs). Over the years, BSR has been severely affecting OP productivity especially in South East Asian countries such as Malaysia and Indonesia^13–15^. A continuous prevalence of BSR suggests that effective disease control strategies are still lacking. The pathogenic fungus penetrates into host cells by using thin-like needle structures, also known as the micro-hyphae^16^ prior to colonization (biotrophic nutrition). During colonization, the host plant remains asymptomatic until the pathogen switches into necrotrophic nutrition. At this stage, white cellulose of the wood component becomes exposed upon the successful degradation of lignin component. Later, the OP trunks form fruiting bodies and eventually rot before death. In large-scale oil palm plantations, disease symptoms of BSR include stunted and unopened spear leaves, yellowing and senescence of upper fronds and ‘one-sided mottling’ of the canopy^17,18^. Likewise, under controlled environment, artificially *G. boninense* challenged oil palm seedlings have shown the emergence of fruiting bodies followed by yellowing and browning of leaves prior to seedling necrotic death^19^.

The plant induced resistance re-conditions host defensive capacity upon elicitation by a specific biotic challenge; systemic acquired resistance (SAR) and induced systemic resistance (ISR). The perception of pathogen by host cells activates a concert of biochemical and metabolic activities which includes the burst of reactive oxygen species, production of secondary metabolites and pathogenesis-related (PR) proteins. These compounds are co-ordinately synthesized and accumulated by host plant for resistance enhancement. The host plant PR genes encode for PR proteins during defense response against microbial attack^20^. These low-molecular weight proteins of about 6–43 kDa in size have been observed in numerous host–pathogen interactions particularly during fungal infection^21^. Both *β-1,3-glucanase* (EC 3.2.1.39) and *chitinase* (EC 3.2.1.14) are PR proteins widely characterized for fungal pathogen resistance enhancement in crop plants. The *β-1,3-glucanase* catalyzes the hydrolytic cleavage of (1,3)-β-D-glucosidic linkages present in (1,3)-β-glucans of fungal cell walls while *chitinase* acts on the breakdown of chitin (β-1,4-linked N-acetyl glucosamine) homopolymer. The involvement of induced resistance has been correlated with the increase activity of PR proteins^22^.

In Southeast Asia region, BSR of OPs is managed mainly by fungicidal treatments. Application of fungicides effectively prolongs the life span of the infected palms and their subsequent productivity over time. In Malaysia, the hexaconazole application is the most preferred method for BSR management as the systemic fungicide is reasonably effective at a low concentration (EC50 = 0.026 µg ml^−1^) and has convenient application strategies such as the soil drenching and soil injection methods^23^. In this study, a BSR disease trial was performed to compare the effect of phenazine and hexaconazole on the expression of PR genes and disease development (disease severity and plant vigour traits). A basal stem rot disease trial consists of *G. boninense* inoculated seedlings treated with phenazine (*P. aeruginosa* derivatives) and hexaconazole was conducted. Root-specific expression of PR genes at the early stage of infection was quantified by reverse-transcriptase PCR to identify involvement of induced resistance in the treated seedlings. Our findings suggest that phenazine isolated from *P. aeruginosa* could be efficiently exploited for biological pesticide formulation as an alternative to chemical pesticides. Phenazine-based biological pesticide would offer an environmentally sound solution to BSR management apart being highly effective for disease suppression and oil palm growth enhancement.

## Results

### Disease assessment of the infected oil palm seedlings

**Figure 1:**
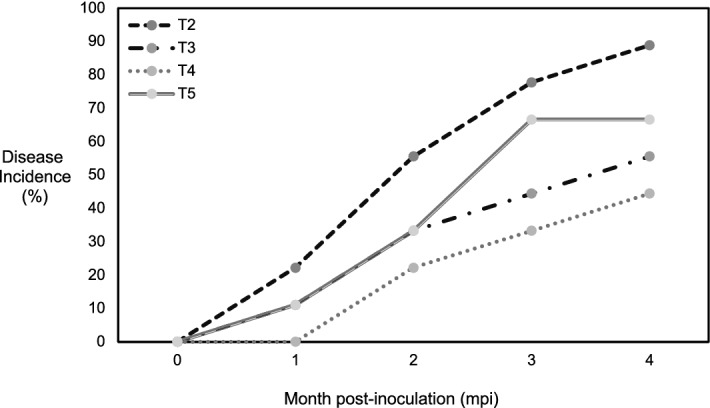
Disease incidence (DI) of oil palm seedlings challenged with *Ganoderma boninense* and treated with phenazine and hexaconazole. T2–T5 denote the different treatments: T2, *G. boninense* inoculated oil palm seedlings; T3, *G. boninense* inoculated oil palm seedlings treated with 1 mg/ml phenazine; T4, *G. boninense* inoculated oil palm seedlings treated with 2 mg/ml phenazine; T5, *G. boninense* inoculated oil palm seedlings treated with hexaconazole. The DI values are recorded at monthly intervals (1–4 mpi). All values are expressed as the means of three biological replicates.

Oil palm seedlings artificially infected with *G. boninense* developed the BSR disease symptoms over a period of 4 months. Apparent foliar symptoms observed includes yellowing followed by browning of lower leaves and formation of sporophore/fruiting bodies. The disease incidence (DI%) computed based on the foliar symptoms gradually increased over the months post-inoculation (mpi). The non-inoculated seedlings from the negative control treatment (T1) showed absence of any visible disease symptoms. Generally, the DI% of the inoculated + treated seedlings (T3, T4 and T5) was lower than the control seedlings (T2) at each mpi. A lower DI corresponds to the effectiveness of the treatment to suppress the disease progression. Among the treatment groups, seedlings inoculated + treated with phenazine at 2 mg/ml (T4) showed the lowest DI value throughout 1-to-4 mpi. Interestingly, at 1 mpi, the DI was zero for T4 in comparison to other treatment groups: T2, DI = 22.22%; T3, DI = 11.11%; T5 DI = 11.11%. At 2 mpi, the DI of each treatment groups increased steeply as following: T2, DI = 55.56%; T3, DI = 33.33%; T4, DI = 22.22%; T5, DI = 33.33%. At 3 mpi, the DI continued to show slight increment at 77.78, 44.45, 33.33 and 66.67 for T2, T3, T4 and T5, respectively. At 4 mpi, the DI of the phenazine treated seedlings showed the lowest values: DI = 44.44%, for T4 and DI = 55.56% for T3. This was followed by the inoculated + hexaconazole treated seedlings (T5) at DI = 66.67% and T2 at DI = 88.89% (Fig. 1).

Further, the basal stem rot disease progression was evaluated by the following parameters: area under the disease progress curve (AUDPC), epidemic rate (ER) and disease reduction (DR). The effectiveness of the treatment to delay or to suppress the onset of BSR symptoms was expressed as DR whereas the ER reflected on the progress of disease development under the different treatment per se. The AUDPC, ER and DR values among the treatments showed significant differences at *p* < 0.05. The AUPDC of the untreated seedlings (T2) was significantly highest at 116.3 followed by the inoculated + hexaconazole treated seedlings (T5) at 86.90. Seedlings inoculated + treated with phenazine at 1 mg/ml showed AUPDC = 77.65 and seedlings inoculated + treated with phenazine at 2 mg/ml showed the least value at AUPDC = 54.93. Similar trend was obtained for the ER scores: Treatment with the least AUPDC scores obtained the smallest ER and vice versa. The ER values of each treatment corroborated to the DR values. Among the treatments, at ER = 0.111 and DR = 52.76, the application of phenazine at 2 mg/ml (T4) showed the best efficiency to control BSR development whereas the application of hexaconazole was found least efficient at ER = 0.1666 and DR = 25.27 (Table 1).

**Table 1:** Effect of phenazine and hexaconazole applications on oil palm seedlings artificially infected with *Ganoderma boninense*.

Treatment	AUDPC^1^	ER^1^	DR^1,2^
**T1 (negative control)—Gb**			
T2 (positive control) + Gb	116.30^a^	0.2222^1a^	0.00
T3 (phenazine 1 mg/ml) + Gb	77.65^c^	0.1388^c^	33.23^b^
T4 (phenazine 2 mg/ml) + Gb	54.93^d^	0.1111^d^	52.76^a^
T5 (hexaconazole 0.048 mg/ml ) + Gb	86.90^b^	0.1666^b^	25.27^c^

Means within columns followed by different letters are significantly different at *p* < 0.05 according to Least Significant Difference (LSD) test. Gb represents the *Ganoderma boninense* inoculum and + /− sign denotes with/without the artificial infection of oil palm seedlings. Each treatment (T1–T5) are described in parentheses: T1 and T2; control samples T3–T5; treated samples.

^1^Means of 3 biological replicates.

^2^% of disease reduction.

### Effect of treatment conditions on oil palm seedlings’ vigor traits

The plant height, stem diameter and chlorophyll content were evaluated at 1-to-4 month post-inoculation (mpi) whilst the plant fresh weight and root biomass was recorded at 4 mpi only. In general, the application of phenazine at both 1 mg/ml (T3) and 2 mg/ml (T4) on *Ganoderma boninense* inoculated oil palm seedlings improved the plant height, stem diameter, plant fresh weight, root fresh weight and root dry weight throughout the disease trial as compared to hexaconazole treated seedlings. The plant height for seedlings subjected to all treatments ranged at 28.9–40.8 cm and increased with the mpi. At 1, 2 and 3 mpi, seedlings treated with phenazine (T3 and T4) showed significantly the highest growth performance at 39.9 and 40.8 cm, respectively. At 4 mpi, the plant height of all treatments showed no significant difference.

Stem diameter increased throughout the disease trial in all treatments at various pattern. The stem diameter of seedlings inoculated + treated with 1 mg/ml and 2 ml/ml phenazine (T3 and T4) was higher than the non-inoculated (T1), inoculated + non-treated (T2) and inoculated + hexaconazole (T5) treated seedlings. The T4 seedlings showed the best performance consistently from 1 to 4 mpi with stem diameter ranging at 5–7.6 cm. The stem diameter of the T4 seedlings was the greatest while the inoculated + hexaconazole seedlings (T5) showed the least growth increment (2.6–4 cm).

All the inoculated + treated seedlings (T3, T4 and T5) showed a higher biomass (plant fresh weight, root fresh weight and root dry weight) in comparison to the inoculated + non-treated seedlings (T2). Plant fresh weight at 4 mpi was significantly highest in the inoculated + phenazine treated seedlings (T4) at 46.9 kg. Root fresh weight was highest in the non-inoculated seedlings (T1) at 17.7 kg whilst the root dry weight was significantly highest in the T4 seedlings. Among the treatments, seedlings inoculated + treated with 2 mg/ml of phenazine (T4) showed the best growth performance and the inoculated + hexaconazole treated seedlings (T5) was the poorest.

**Figure 2:**
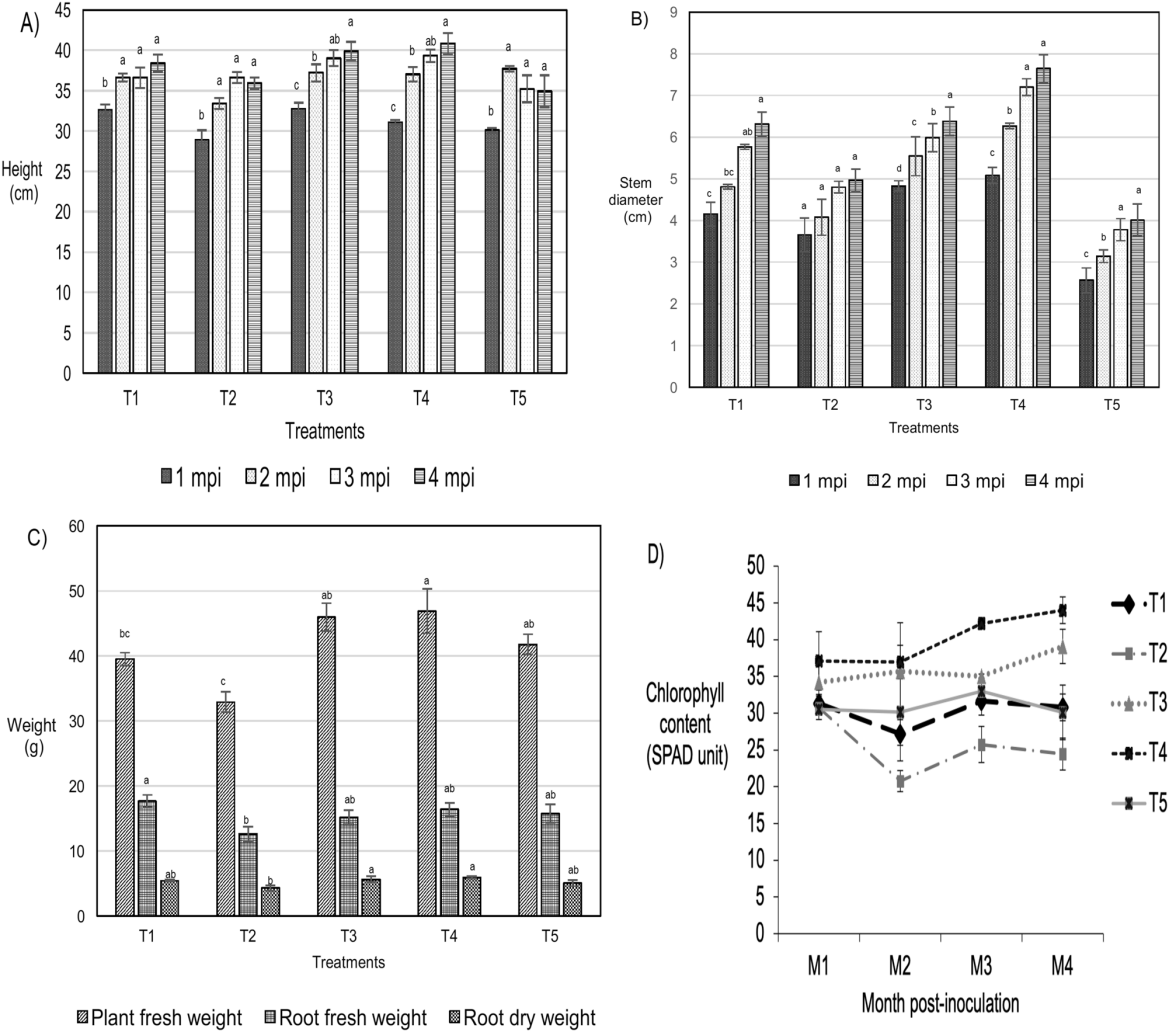
Plant vigour assessment of oil palm seedlings under various treatments under a disease trial: T1; non-inoculated oil palm seedlings without application, T2; *Ganoderma boninense* inoculated oil palm seedlings without application, T3; *G. boninense* inoculated oil palm seedlings treated with 1 mg/mL of phenazine, T4; *G. boninense* inoculated oil palm seedlings treated with 2 mg/ml of phenazine, and T5; *G. boninense* inoculated oil palm seedlings treated with 1 mg/ml hexaconazole. Treatments were evaluated at monthly intervals from 1-to-5 month post-inoculation (mpi). (**A**) Height, (**B**) stem diameter, (**C**) weight and (**D**) chlorophyll content expressed in SPAD units. All values are indicated as average means of three biological replicates and error bar represents the standard error.

Chlorophyll content of the non-inoculated seedlings (T1) ranged from 27.2 to 31.6 throughout the disease trial. At 1 mpi, the chlorophyll content of the inoculated seedlings (T2) showed the least value at 30.7, followed by an apparent decline at 2 mpi (20.8) and slight increment in the subsequent sampling intervals (3 and 4 mpi). The results of T2 may suggest that disease development progressively reduced the chlorophyll content. For seedlings inoculated + treated with phenazine (T3 and T4), the chlorophyll content increased gradually with the sampling intervals: T3; 34.2–39.1 and T4; 37–44. The seedlings inoculated + treated with hexaconazole (T5) showed no apparent trend: decreased at 2 mpi (30.2) followed by an increase (33.0) at 3 mpi and a decrease (30.1) at 4 mpi (Fig. 2).

### Histological analysis of the non-treated and phenazine treated root tissues

**Figure 3:**
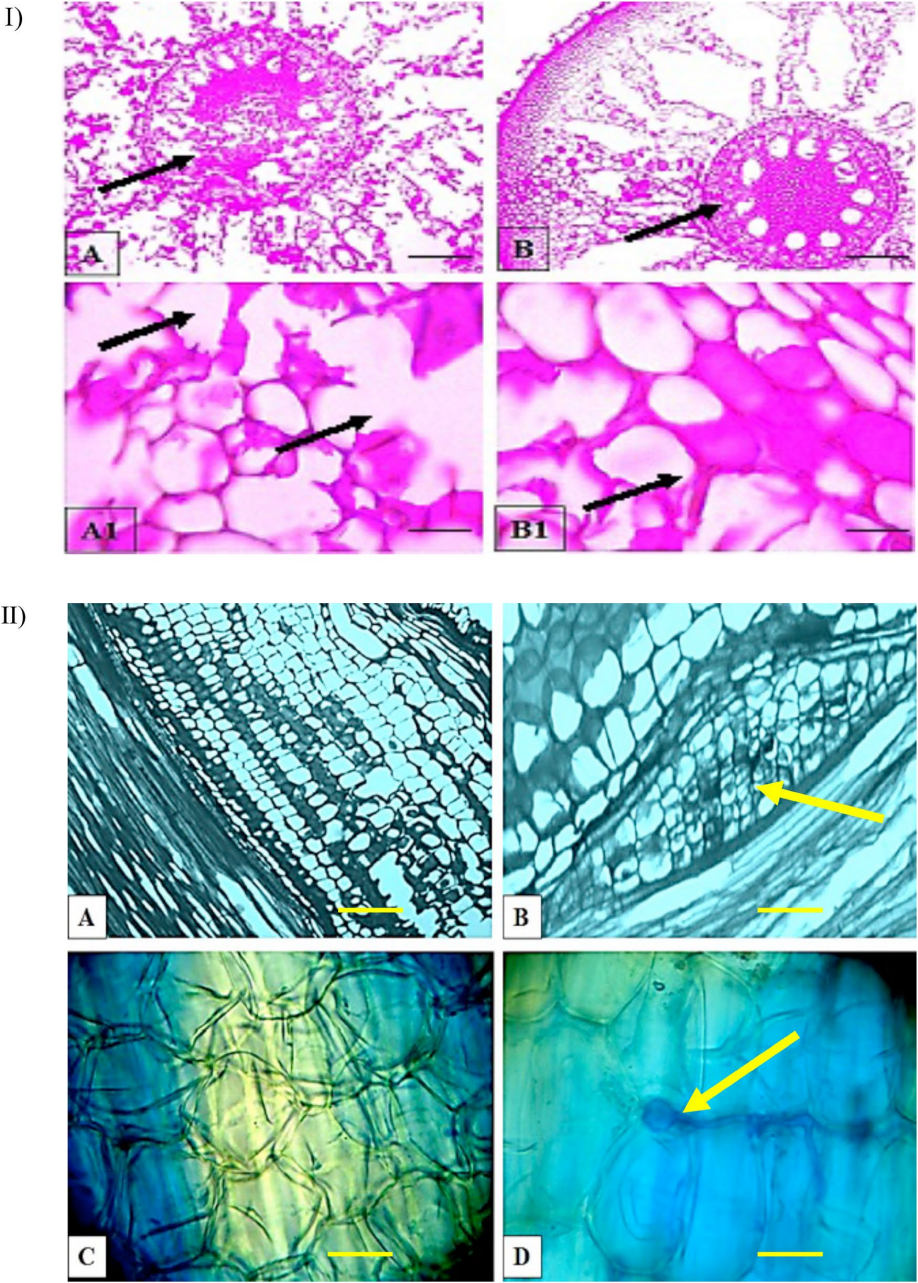
Histological observation of control and infected (at 4 month post-inoculation) oil palm root tissues. (**I**) Horizontal sections (**A**) Infected root tissues showing degraded vascular bundle (black arrow) (× 10 magnification), (**A1**) disintegrated parenchyma cells (black arrow) ( × 40 magnification). (**B**) Phenazine treated root tissues showing an intact vascular bundle (black arrow) ( × 10 magnification) and (**B1**) parenchyma cells (black arrow) ( × 40 magnification). (**II**) Longitudinal sections. (**A**) Healthy root tissue ( × 10 magnification), (**B**) infected root tissue showing Ganoderma colonization evident by formation of bulb like structure ( × 10 magnification) (yellow arrow), (**C**) phenazine treated root tissue ( × 40 magnification), and (**D**) Appressorium (yellow arrow) penetrating into cortex region ( × 40 magnification). Scale bars are equivalent to 30 µm.

A histological analysis was conducted on root tissues obtained from the inoculated + non-treated and inoculated + phenazine treated oil palm seedlings (4 month post-inoculation). All cross-sections of the seedlings showed three distinct layers which include the epidermis (outermost), cortex (middle) and vascular bundle (inner-most). The inoculated + phenazine treated root tissues were found healthy in comparison to the inoculated + non-treated seedlings. The inoculated + non-treated seedlings showed clear formation of disintegrated parenchyma cells at the vascular bundle while the inoculated + treated seedlings had intact cells, free from any form of malformations (Fig. 3).

### Phenazine application induces the expression of pathogenesis-related (PR) genes

**Figure 4:**
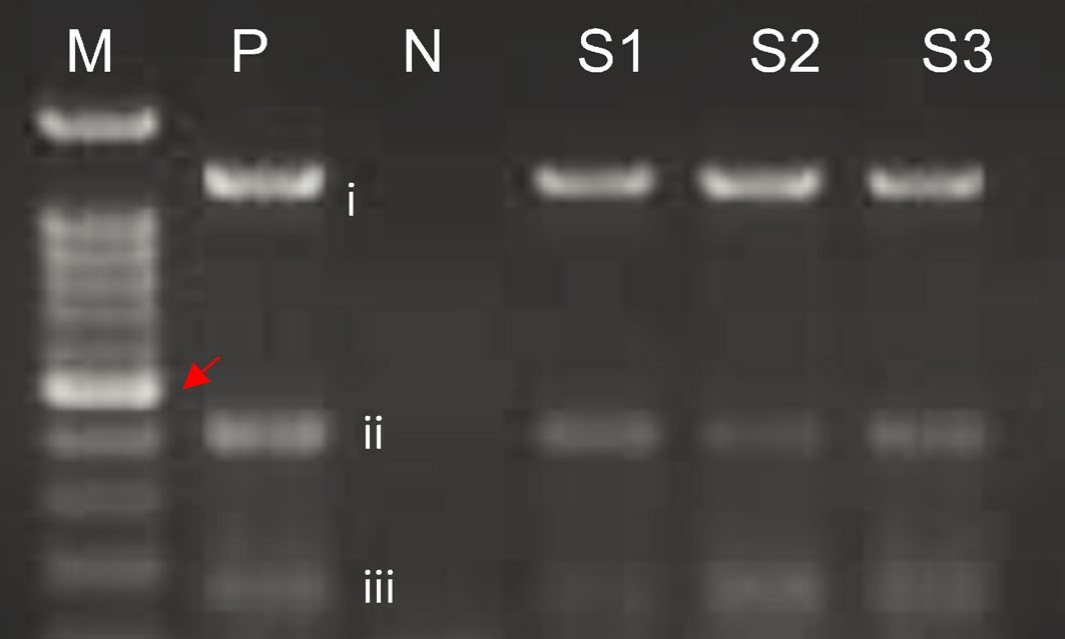
Amplification of pathogenesis related genes from root tissues of oil palm seedlings: *chitinase* (i), *β- 1,3 glucanase* (ii) and a housekeeping *GAPDH* gene (iii). Red arrow indicate 500 bp. Each lane is labelled as following: M; 100 bp DNA ladder (marker), P; positive control comprised of PCR product of target gene at the following sizes: (i) 891 bp, (ii) 491 bp and (iii) 100 bp, N; negative control and S = samples at 4 weeks-post-inoculation S1; *G. boninense* inoculated + phenazine 1 mg/ml treated oil palm seedlings, S2; *G. boninense* inoculated + phenazine 2 mg/ml treated oil palm seedlings and S3; *G. boninense* inoculated + hexaconazole treated oil palm seedlings.

The expression of PR genes (chitinase and β-1,3 glucanase) were found to be up-regulated in oil palm root tissues with the presence of *G. boninense* and phenazine application throughout the following sampling period: 2, 4, 6, and 8 weeks post-inoculation (wpi). The PCR product of *chitinase* and *β-1, 3 glucanase* genes from oil palm seedlings were 820 bp and 419 bp, respectively (Fig. 4). For quantification of PR genes, the relative quantification of the corresponding to target PCR products was calculated against a housekeeping gene, the GADPH 109 bp. Since the identification of two PR gene products along with house-keeping gene product were done simultaneously in a PCR cocktail, formation of primer dimer was observed during the gel view (S3). In this study, a relative expression level of > 2 was assumed significant.

**Figure 5:**
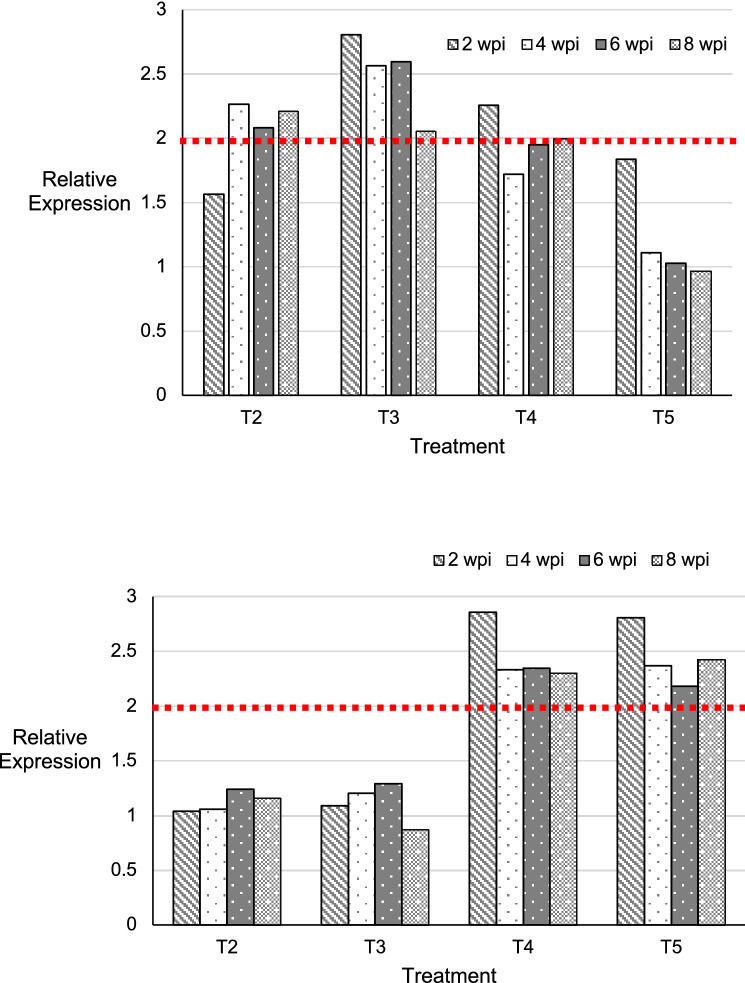
Relative quantification of *chitinase* (top) and *β-1,3 glucanase* (bottom) gene in oil palm root tissues subjected to different treatment condition using RT-PCR method: T2; *Ganoderma boninense* inoculated oil palm seedlings, T3; *G. boninense* inoculated + phenazine 1 mg/ml treated oil palm seedlings, T4; *G. boninense* inoculated + phenazine 2 mg/ml treated oil palm seedlings and T5; *G. boninense* inoculated + hexaconazole 1 mg/ml treated oil palm seedlings. Bars with different patterns represent the following sampling intervals: 1, 2, 3 and 4 weeks post-inoculation (wpi). Red dotted line at relative expression > 2 indicates significant difference.

In the inoculated seedlings (T2), the expression level of *chitinase* was low at 2 wpi (1.56) before a gradual increase of > 2 in the subsequent sampling periods (4, 6 and 8 wpi). Among the treated seedling groups, only the inoculated + phenazine (1 mg/ml) treated seedlings (T3) showed a significant up-regulation of *chitinase* gene at all sampling intervals (2, 4, 6 and 8 wpi). The expression level was highest at 2 wpi (2.8) and decreased at 8 wpi (2.1). The inoculated + hexaconazole treated seedlings (T5) showed the lowest expression levels throughout the sampling periods. At 2 wpi, the expression level was 1.84 declined gradually to 1.11, 1.03 and 0.97 at 4, 6, 8 wpi, respectively (Fig. 5).

The expression of *β-1,3 glucanase* at 2, 4, 6 and 8 wpi was significant at > 2 in the inoculated + phenazine (2 mg/ml) treated seedlings (T4) and inoculated + hexaconazole treated seedlings (T5). The *β-1,3 glucanase* expression was highest at 2 wpi at 2.86 and 2.81 in T4 and T5, respectively. The expression pattern in the inoculated seedlings (T2) increased from 2-to-6 wpi followed by a decline at 8 wpi (1.15). Similar trend was observed in the inoculated + phenazine (1 mg/ml) treated seedlings (T3); expression pattern increased (1.09–1.30) from 2-to-6 wpi before a decline at 8 wpi (0.87) (Fig. 5).

## Discussion

World production and consumption of oils and fats have been growing steadily over the decades; a 4% annual increase over the past 32 years^24^ with palm oil among them. The palm oil has been extensively marketed for the biofuel industry, forage feed and in the production of a wide range of chemical products such as the soaps and cosmetics. An oil palm tree grows well in a hot climate and has an average life span of 25–30 years. The tree is able to yield one tonne of oil on about 0.26 hectares of land; about 8–10 folds higher than soybean, sunflower and rapeseed. In the South-east Asian region, the oil palm industry shapes the social-economic development of the nation with an annual export revenue of 65 billion. The highly efficient oil-seed crop is affected by numerous diseases; basal stem rot (BSR), leaf spot disease, algae leaf spot, fruit rot, stem wet rot and charcoal base rot^25^. Amongst these field emerging diseases, the BSR of oil palms has gained enormous attention as significant yield losses are ongoing and reliable control strategies remain scarce. In Malaysia, the oil palm covers about 5.85 million hectares and represents 60% of the nation’s agricultural land. In a survey conducted throughout 1995–2017, the BSR disease incidence reported a gradual increase from 1.51% (1995) to 3.17% (2012) and to 7.4% (2017). The BSR disease incidence was affected by oil palm age, planting density and soil properties^25,26^.

The BSR of oil palms is caused by the basidiomycetous *Ganoderma boninese*, a hembitrophic white fungus which colonizes the host by biotrophic nutrition during the early stage infection and later evolves into a necrotroph as the disease progresses to cause host death^19^. The pathogen spreads the disease through root contact between healthy and infected tissue and by a vector species; *Episcapha 4-maculata* (Episcapha/tiger beetle) carries the *G. boninense* basidiospores in its mouthparts^26^. To date, BSR management tools include sanitation/cultural practices, application of fungicides, fumigants and biocontrol agents. Popularly employed fungicides applied via the hand-knock injector and trunk drenching methods are dazomet, hexaconazole, thiram, benomyl, triadimefon, triadimenol, tridemorph and tetraconazole^24^. Among these fungicidal treatments, the application of hexaconazole for *G. boninense* diseased palm trees has demonstrated the best efficiency as the infected palms could remain fertile upon a prolong use^27,28^. The hexaconazole is a member of the triazole group which carries the systemic demethylation inhibitors that acts selectively to impede growth of the fungal tissues; vegetative mycelium^29,30^. However, with a continuous application of fungicide, the notorious *G. boninense* evolves to acquire resistance. Alternative approaches are increasingly sought to replace the use of fungicides which are becoming inefficient over a long term.

Plant-beneficial pseudomonads are excellent biocontrol agents for the fungal pathogen inhibition and subsequent disease suppression. *Pseudomonas* spp. are able to promote plant growth and suppress soil-borne diseases in many economically important crops^31^. The antifungal phenazine compounds secreted by *Pseudomonas* spp. are pivotal determinants for the suppression of soil-borne diseases. A large number of *Pseudomonas* spp. such as the *Pseudomonas chlororaphis*^32^, *Pseudomonas putida*^33^, *Pseudomonas fluorescens*^34,35^ were shown to positively impact the suppression of root take-all disease of wheat and barley, chrysanthemum yellows phytoplasma infection of daisy and Fusarium wilt of radish. Similarly, the *Pseudomonas aeruginosa* 7NSK2 showed induced resistance to *Botrytis cinerea* infecting beans^36^. In a study conducted on oil palms, the positive effects of plant growth promoting rhizobacteria (PGPR) on plant overall growth and development has shown remarkable impact on the root morphology: significant increase in lateral root length, root hair number, shoot length and yield. The application of endophytic bacteria evident both an improved vegetative growth and suppression of BSR in oil palm seedlings^37^ In others, the bacterial endophytes *P. aeruginosa* strain UPMP3 independently increased oil palm seedlings’ growth^38^ and effectively suppressed the BSR incidence^39,40^. The *P. aeruginosa* UPMP3 suppresses *G. boninense* infection via two distinct ways. First, the fungal penetration into host is blocked through antibiosis establishment by *P. aeruginosa* found colonizing the root tissues. Later, the induction of defence-related metabolites in host aids the hydrolyses of the *G. boninense* cell wall and subsequently destroy the invading pathogen^38,41^.

Under a controlled environment, various factors collectively contribute to the success of *P. aeruginosa* UPMP3 colonization onto oil palm roots. In comparison to fungicides, the direct application of endophytes onto oil palm roots aimed for priming the plants is certainly a cost-effective measure because formulation procedure could be omitted virtually. However, the efficiency for these endophytes to establish their population and remain stable under adverse environmental conditions is highly uncertain. The *P. aeruginosa* UPMP3 thrives well in rhizosphere planes subjected to an optimal light, temperature, and soil physico-chemical properties, all of which that does not describe the real condition of any oil palm plantations. In Malaysia, endophytes-based commercial augmentative biological control agents for BSR are available. Nevertheless, the efficiency of these biocontrol agent, especially *P. aeruginosa* UPMP3-based is limited by the following constraints: (1) may pose risk to human health as *P. aeruginosa* is an opportunistic human pathogen, (2) the population establishment of *P. aeruginosa* in the soil rhizosphere fluctuates with soil properties, and (3) population size/fitness of the *P. aeruginosa* is affected by weather. With climate change followed by an unpredictable weather pattern, application of *P. aeruginosa* UPMP3 endophytes for disease management becomes particularly difficult since the bacterial population demands an optimal condition for growth, sustenance and subsequent mode of action.

In this study, phenazine extracted from *P. aeruginosa* UPMP3 played a dual role on the overall physiology of oil palm seedlings: (1) suppressed BSR development and, (2) promoted plant growth. Application of phenazine onto oil palm root tissues reduced the BSR disease incidence significantly throughout the disease trial. The seedlings treated with phenazine (T4: *G. boninense* inoculated + phenazine at 2 mg/ml) showed the lowest disease score, at AUDPC = 54.93 unit. Application of naturally synthesized phenazine was far more effective than fungicide (T5: *G. boninense* + hexaconazole) which scored AUDPC = 86.90 units. Phenazine was applied at two different concentrations on a monthly basis and our results suggest that BSR suppression is most effective at a concentration of 2 mg/ml. The expression of chitinase and β-1,3 glucanase, hydrolytic enzymes which act on fungal cell wall components such as the glucan, chitin and peptidoglycans is an indicator of invoked systemic resistance in the host tissues. In the untreated plants, the BSR disease symptoms become more severe as the expression levels of chitinases and *β-1,3 glucanase* (T2: positive control + *G. boninense*) decline. An increase in the chitinase and *β-1,3 glucanase* gene expression levels were probably a general response of host plant to fungal contact, irrespective of its nature; beneficial or pathogenic. In oil palm-*G. boninense* treated with phenazine, an up-regulated expression of chitinase and *β-1,3 glucanase* genes indicated induced oil palm defense responses. These results suggest that chitinases alone or in combination with *β-1,3-glucanase*, may play a significant role in limiting *Ganoderma* infection of oil palm root tissues. In the present study no external disease symptoms were observed up to 3 months of the antibiotic treatment, while at the end of 4 months the DI and DS percentage were found to be significantly lower than the positive control (plants treated with *G. boninense*) and the hexaconazole treated plants. The application of phenazine demonstrated bio-fertilizing ability as the stem diameter of the phenazine treated seedlings (T3 and T4) were significantly higher than the Ganoderma-inoculated alone seedlings (T2). Application of hexaconazole (T5) inhibited growth of the stem diameter as the T5 was significantly lower than T2. These findings may suggest that the ability of hexaconazole to supress basal stem rot disease is slightly traded off for the optimal growth of stem diameter.

Phenazine is an antibiotic metabolite which is produced by the microbial population after reaching a threshold level^42,43^. Further studies on the quorum-sensing phenomenon of *P. aeruginosa* UPMP3 along with genomic information on gene sequences with potentials for manipulation via genetic engineering is required for large-scale phenazine biosynthesis and production. To our knowledge, this is the first study to demonstrate that phenazine extract from *P. aeruginosa* can alter the transcriptional activity of PR-related genes in oil palm-*Ganoderma boninense* pathosystem. The phenazine extract holds promising potentials in controlling basal stem rot disease development and oil palm growth enhancement. Displaying both the bio-fertilizing and biocontrol capabilities, future strategies directed towards commercialization of phenazine extract is necessary for basal stem rot management. Additional studies are required to optimize the timing of applications while making sure that the application protocol is fully compatible with the standard oil palm agricultural practices.

## Material and methods

### Planting materials

A total of 120 3-month-old oil palm seedlings (Dura × Pisifera) were obtained from Sime Darby Seeds and Agricultural Services Sdn. Bhd. (Banting, Malaysia). The seedlings were transferred into polyvinyl bags (10 cm × 10 cm) loaded with approximately 3 kg of soil mixture. The soil mixture (soil: peat: sand at 3:2:1) purchased from Miba-Mansura Trading Sdn Bhd, Selangor, Malaysia was sterilized for 2 h at 100 °C and allowed to cool for 3 days before use. All transplanted seedlings were watered on a daily basis and supplementary fertilizers were not applied throughout the experiment.

### Experimental design

The experiment was conducted in glasshouse 1C, Field 10, Universiti Putra Malaysia. The seedlings were arranged under a completely randomized design (CRD) with five different treatments represented as three biological replicates, each. Description on each treatment is given in Table 2.

**Table 2:** Treatment conditions of the *Ganoderma boninense-*inoculated oil palm seedlings.

Treatment	Description
T1	Oil palm seedlings (negative control)
T2	*G. boninense* inoculated seedlings (positive control)
T3	*G. boninense* inoculated seedlings + phenazine (1 mg/ml)
T4	*G. boninense* inoculated seedlings + phenazine (2 mg/ml)
T5	*G. boninense* inoculated seedlings + hexaconazole (0.048 mg/ml)

Each treatment is represented by three biological replicates.

### Inoculum preparation and artificial infection of oil palm seedlings

*Ganoderma boninense* PER71 was grown on malt extract agar (MEA) at 28 °C. A 7-day-old mycelium culture was used for large-scale preparation of *G. boninense* inocula, hereafter termed *Gano*-wood block inocula. Freshly cut rubber (*Hevea brasiliensis*) wood blocks (RWBs) measuring 6 cm × 6 cm × 6 cm were dried at 80 °C for 3 days, washed briefly in tap water and soaked in distilled water for an overnight. The RWBs placed in polypropylene bags were autoclaved at 121 °C for 20 min. Approximately 100 ml of molten MEA was poured into each bag containing the RWB*.* The bags were closed by drawing its open end through a polyvinyl chloride (PVC) tubing (4 × 2 cm) and the remaining hole was plugged with cotton wool. The tubings of the polypropylene bags were covered with aluminium foil and the bags were autoclaved again for 30 min at 121 °C. Following a rest period for an overnight at room temperature, each bag of the RWB was inoculated with five plugs (5 mm diameter) of *G. boninense* mycelium and left for incubation in a dark chamber at 28 ± 2 °C. After 4 weeks, only *Gano*-wood block inocula that were fully covered with mycelia were selected for artificial infection of oil palm seedlings under the disease trial study (Fig. s1).

The oil palm seedlings were inoculated with *Gano*-wood block inocula as described by Khairuddin^44^. Briefly, oil palm seedlings were carefully excavated from the soil. The bole of the oil palm seedling was positioned directly on the *Gano*-wood block inoculum. The root tissues were dispersed around the sides of the *Gano*-wood block. Finally, the oil palm seedling seated onto the *Gano*-wood block was firmly re-planted into the soil (Fig. s2). At 2 week post-inoculation, each oil palm seedling from the relevant treatment groups, received 20 ml of phenazine and hexaconazole as described in Table 2 for three subsequent months. All applications were performed according to the soil drenching method (Fig. s3).

### Large-scale isolation of phenazine from ***Pseudomonas auroeginosa*** UPMP3

The phenazine was isolated from *P. aeruginosa* UPMP3 population according to Chang and Blackwood’s method^7^ with slight modifications. The bacterial (approximately 10^8^ cells/ml) population was inoculated onto King’s B broth and incubated at 30 °C with shaking at 150 rpm for 2 days. Following the bacterial exponential growth, cells were centrifuged at 3,500 rpm for 7 min. The resultant pellet was suspended into pigment production medium broth (5 ml) and re-incubated at 30 °C with shaking at 150 rpm for 4 days. The culture was adjusted to pH 2.0 by using concentrated HCl before extraction with an equal volume of benzene (analytical grade). The benzene layer was evaporated at 40 °C and the resultant residue was suspended into 1 ml of absolute methanol. To further confirm and quantify the isolated phenazine, a HPLC analysis was performed on a HPLC system (Waters) according to Watson et al. 2005 with the following modifications: the HPLC system was coupled to a 150 × 4.6 mm Ultracarb 5 µm ODS (30) column and solvent A (water: trifluoroacetic acid, 100:0.04, v/v) and solvent B (acetonitrile: water: trifluoroacetic acid, 90:10:0.04, v/v/v) were applied throughout the run at a sample injection volume of 10 μl. Solvent B was set as the mobile phase and the following condition was applied: solvent A was maintained for 15 min, followed by 90% solvent A and 10% solvent B solution for another 10 min. Next, a linear gradient of 70% solvent A and 30% solvent B solution was applied for 15 min before changing it to 64% solvent A and 34% solvent B solution, maintained till the end of the run (total time: 65 min). Phenazine was identified and quantified using authentic antibiotic standards prepared at various concentration. All samples were analysed thrice and concentration of phenazine antibiotics in each sample were expressed as μg/ml.

### Disease assessment: plant vigour traits, disease incidence and disease severity

To determine the effect of the treatments on plant growth and disease suppression, a total of four sampling points was fixed throughout the disease trial; 1, 2, 3, and 4 month post-inoculation (mpi). At each sampling point, the following parameters were recorded: plant height, stem diameter, plant fresh weight, and root fresh weight, dry weight. Height was measured from one cm above the soil level (ground) to the tip of the leaves using a tape. The stem diameter was measured using a vernier digital calliper. Chlorophyll content was recorded at three random positions of a fully developed leaf in each seedling using a chlorophyll meter (SPAD-502-Minlota Co. Ltd. Japan). Plant fresh weight and root fresh weight were measured using a weighing scale while the dry weight was quantified similarly after drying at 50 °C for 72 h.

BSR disease development was recorded at each mpi according to a modified disease assessment scale by Breton et al.^45^. Symptoms that appear on the foliage were scored as following: 0, healthy plants show no visible symptoms; (1) yellowing of at least > 2 lower leaves and/or rhizomorph formation at the base of the bole; (2) necrosis of at least > 2 lower leaves with button-like sporophore at the base of the bole; (3) > 50% necrosis of leaves and sporophore at the base of the bole and (4) seedling complete necrosis and sporophore at the base of the bole. Symptoms on the internal root tissue were scored as following: (0) Healthy seedlings with no visible sign of internal rot; (1) 20% rotting of tissues; (2) 20–50% rotting of tissues; (3) > 50% rotting of tissues; (4) > 90% rotting of tissues. Foliar symptom scale was used to compute disease severity (external) according to Liu^46^ whereas the root internal symptoms scale was used to calculate internal disease severity according to Breton^45^ with slight modifications. For disease incidence (DI) % computation, the infected seedlings were described as following: chlorosis and/or necrosis of leaves, with or without production of sporophore and/or fruiting bodies. The DI% values of all the treatment groups at each mpi were computed to generate a disease progression curve according to Campbell and Madden^47^.

### Histological analysis compares oil palm root tissues under different treatments

To understand the effect of the different treatments (phenazine and hexaconazole) on fungal pathogen colonization, root samples were collected at 4 mpi and prepared for histopathological views. Each root sample was fixed in FAA solution [70% ethanol: 5% glacial acetic acid: 5% formaldehyde at the ratio of 18: 1: 1 (v/v/v)] for 24 h at room temperature. Fixed tissues were dehydrated with a series of ethanol and butanol solutions and embedded in paraffin. Serial sections (10 μm) were performed using a rotary microtome (Leica RM2235, Germany) with a steel knife. The sections were floated in water drops, dried on a hot plate (37 °C), stained with acid fuchsin (0.1%), then counter stained with toluidine blue (0.05%). All steps used which include fixation, dehydration, embedding, sectioning staining and mounting of the root samples were carried out prior to observation under the light microscope (Motic BA300, China)^48^.

### Quantification of pathogenesis-related (PR) genes by semi-quantitative reverse transcriptase PCR

Sampling time points for the quantification of PR-gene products were set as followings: 2, 4, 6, and 8 week post-inoculation (wpi). A total of three biological replicates were used to represent each treatment. At each sampling time point, the root tissues collected were washed thoroughly and ground into fine powder in liquid nitrogen. Total RNA was isolated according to the manufacturer’s protocol (Total RNA Mini Kit (Plant), Geneaid Biotech Ltd., Taiwan). The concentration and quality of the total RNA and subsequent cDNA yield was determined by Nanodrop 2000 cc (Thermo Scientific, USA). The integrity of the total RNA was evaluated using agarose gel electrophoresis. High quality total RNA samples were treated with DNase 1 (Fermentas, UAB, Lithuania) according to the manufacturer’s guideline before the first-strand cDNA synthesis. First-strand cDNA was synthesized using the RevertAid First strand cDNA synthesis kit (Thermo Scientific, USA) following the manufacture’s instruction.

The amplifications of *chitinase*, *β-1, 3 glucanase* and a housekeeping [*glyceraldehyde phosphate dehydrogenase* (*GAPDH*)] genes were performed in a PCR cocktail described as following: 2X PCR Master Mix (Fermentase, UAB, Lithuania), 100 ng cDNA, 1X PCR Master Mix, 1 μM primer oligonucleotides and nuclease free water. The primer pairs were designed according to Sathyapriya et al*.*^44^. Amplification was performed in a thermocycler (T3 Thermocycler, Biometra, Germany) with the following conditions: pre-denaturation for 2 min at 95 °C, followed by 40 cycles of denaturation (30 s) at 95 °C, annealing (30 s) at 56 °C, elongation for 1 min at 72 °C and final extension for 10 min at 72 °C. Target gene sequences and their corresponding amplicon products (bands) were viewed by a gel documentation system (Gel Doc 2000, BIO-RAD) after an electrophoresis at the following condition: 2% (w/v) agarose gel immersed in 1X TAE buffer subjected to 65 V for 45 min runtime. The intensity of each band was computationally scored by Gene Tool Software version 1.0 (Quality One 1-D Analysis Software, Version 4.6.9, BIO-RAD) (Fig. s4). The relative expression of each target gene was calculated as shown below:

Digital expression value of house-keeping gene = Digital band intensity value of house-keeping gene/expected amplicon length.

Digital expression value of target gene of interest (goi) = Digital band intensity value of target gene/expected amplicon length/Digital expression value of house-keeping gene.

Relative expression ratio of target gene = Digital expression value of infected (goi) plant/Digital expression value of non-infected control (goi) plant.

### Statistical analysis

For normalization of the raw data, the disease incidence and disease severity values were subjected to arcsine transformation whereas the plant vigour data were subjected to log transformations. A one-way ANOVA was performed and any absence/presence of significant differences between the treatments for the different parameters in this study were determined by Least Significant Difference (LSD) at *p* < 0.05 using the SAS 9.3 statistical package.

## Supplementary information

Supplementary Figure s1.

Supplementary Figure s2.

Supplementary Figure s3.

Supplementary Figure s4.
