# Supplementary material for: Phenazine from Pseudomonas aeruginosa UPMP3 induced the host resistance in oil palm (Elaeis guineensis Jacq.)-Ganoderma boninense pathosystem
Source: Sci Rep. 2020 Sep 24;10:15621. doi: 10.1038/s41598-020-72156-7 (PMC7518433; doi:10.1038/s41598-020-72156-7)
Supplement: Supplementary file 2 — Supplementary Figure s2. [file 41598_2020_72156_MOESM2_ESM.pdf]

Phenazine from *Pseudomonas aeruginosa* UPMP3 outperforms hexaconazole for induced resistance in oil palm (*Elaeis guineensis* Jacq.)-*Ganoderma boninense* pathosystem

Waheeda Parvin[1, 2*], Nisha Govender[3], Radziah Othman[4], Hawa Jaafar[5], Mahbubur Rahman[2, 6] and Mui-Yun Wong[1, 7*]

[1]Department of Plant Protection, Faculty of Agriculture, Universiti Putra Malaysia, Serdang, Malaysia.
[2]Bangladesh Forest Research Institute, Chittagong, Bangladesh
[3] Institute of Biology Systems (INBIOSIS), Universiti Kebangsaan Malaysia, Bangi, Malaysia
[4]Department of Land management, Faculty of Agriculture, Universiti Putra Malaysia, Serdang, Malaysia
[5]Department of Crop Science, Faculty of Agriculture, Universiti Putra Malaysia, Serdang, Malaysia
[6]Department of Biochemistry, Faculty of Biotechnology and Biomolecular Sciences, Universiti Putra Malaysia, Serdang, Malaysia
[7] Institute of Plantation Studies, Universiti Putra Malaysia, Serdang, Malaysia

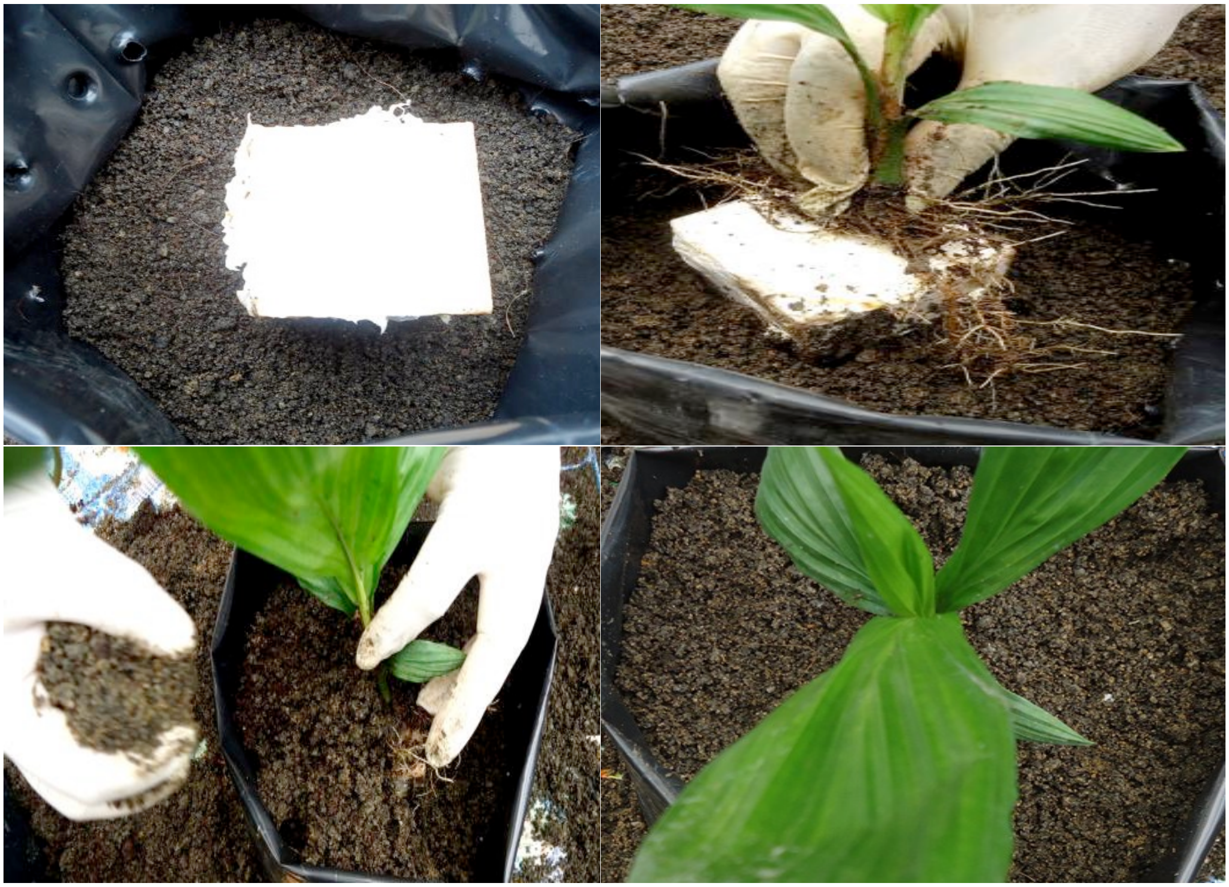

Supp 2: Artificial inoculation of *Ganoderma boninense* colonized rubber wood blocks (RWBs) onto oil palm seedlings. I) Rubber wood block colonized by *G. boninense* is placed in a polybag (approximately 10 cm from the base of bag) filled with soil. II) The oil palm bole is placed upright on the RWB and roots are set in contact with the inocula. III) The RWB together with oil palm seedling is covered with soil mixture. IV) The oil palm seedling is secured with a final weight of 3 kg soil mixture/polybag.
