# Supplementary material for: Phenazine from Pseudomonas aeruginosa UPMP3 induced the host resistance in oil palm (Elaeis guineensis Jacq.)-Ganoderma boninense pathosystem
Source: Sci Rep. 2020 Sep 24;10:15621. doi: 10.1038/s41598-020-72156-7 (PMC7518433; doi:10.1038/s41598-020-72156-7)
Supplement: Supplementary file 4 — Supplementary Figure s4. [file 41598_2020_72156_MOESM4_ESM.pdf]

Phenazine from *Pseudomonas aeruginosa* UPMP3 outperforms hexaconazole for induced resistance in oil palm (*Elaeis guineensis* Jacq.)-*Ganoderma boninense* pathosystem

Waheeda Parvin[1, 2*], Nisha Govender[3], Radziah Othman[4], Hawa Jaafar[5], Mahbubur Rahman[2, 6] and Mui-Yun Wong[1, 7*]

[1]Department of Plant Protection, Faculty of Agriculture, Universiti Putra Malaysia, Serdang, Malaysia.
[2]Bangladesh Forest Research Institute, Chittagong, Bangladesh
[3] Institute of Biology Systems (INBIOSIS), Universiti Kebangsaan Malaysia, Bangi, Malaysia
[4]Department of Land management, Faculty of Agriculture, Universiti Putra Malaysia, Serdang, Malaysia
[5]Department of Crop Science, Faculty of Agriculture, Universiti Putra Malaysia, Serdang, Malaysia
[6]Department of Biochemistry, Faculty of Biotechnology and Biomolecular Sciences, Universiti Putra Malaysia, Serdang, Malaysia
[7] Institute of Plantation Studies, Universiti Putra Malaysia, Serdang, Malaysia

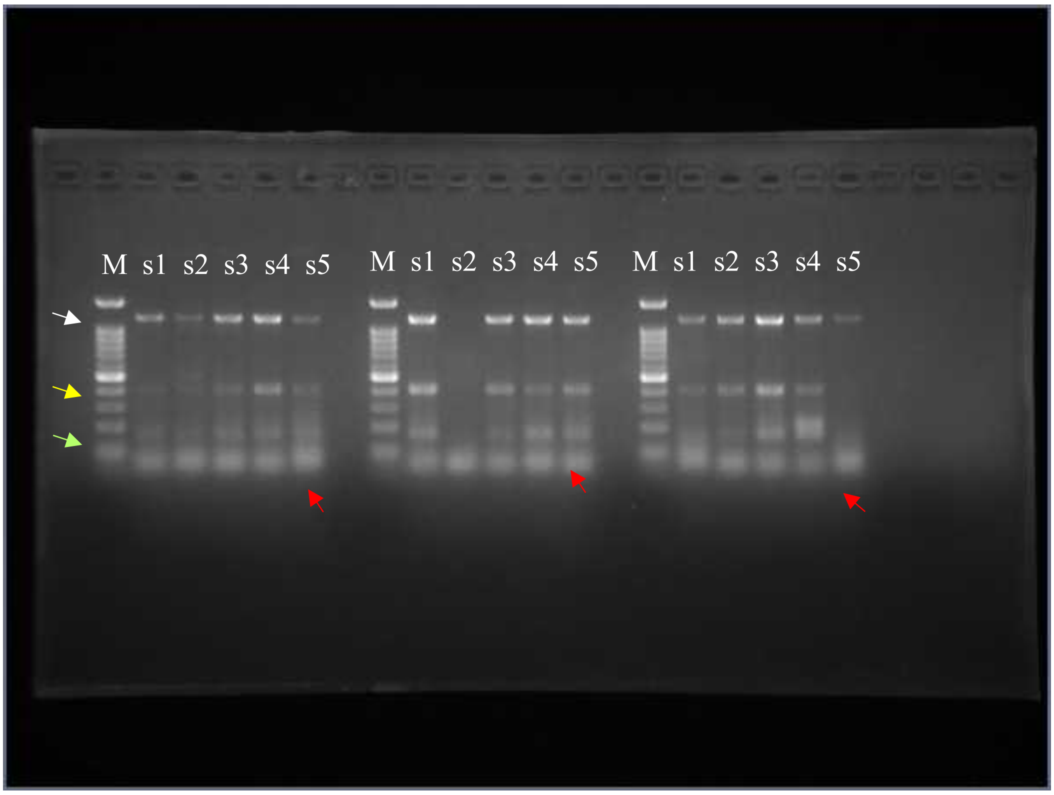

Supp 4: PCR amplification of pathogenesis related genes (*chitinase* and *β- 1,3 glucanase*) and a house keeping gene (*glyceraldehyde phosphate dehydrogenase*) from oil palm root tissues challenged against *Ganoderma boninense.*M denotes a 100 bp DNA ladder marker and each individual lane (s1-s5) with bands denotes sample: root tissues subjected to various treatment conditions. Yellow and green arrows show *chitinase* and *β-1,3 glucanase* gene amplicons, respectively at 820 and 419 bp. Green arrow shows the *glyceraldehyde phosphate dehydrogenase* gene amplicon at 109 bp and the red arrows indicate formation of primer-dimer.
